# 脑脊液循环肿瘤DNA在非小细胞肺癌柔脑膜转移中的临床应用进展

**DOI:** 10.3779/j.issn.1009-3419.2024.102.16

**Published:** 2024-05-20

**Authors:** LI Yubin, LI Xiaoyan

**Affiliations:** 100070 北京，首都医科大学附属北京天坛医院肿瘤内科; Department of Oncology, Beijing Tiantan Hospital, Capital Medical University, Beijing 100070, China

**Keywords:** 肺肿瘤, 柔脑膜转移, 脑脊液, 循环肿瘤DNA, Lung neoplasms, Leptomeningeal metastasis, Cerebrospinal fluid, Circulating tumor DNA

## Abstract

柔脑膜转移（leptomeningeal metastasis, LM）是恶性肿瘤的一种致死性并发症，其在非小细胞肺癌（non-small cell lung cancer, NSCLC）患者中的发生率为3%-5%。LM诊断难、预后差、治疗手段有限且无统一的疗效评价标准，是NSCLC诊疗的难点。循环肿瘤DNA（circulating tumor DNA, ctDNA）由肿瘤细胞脱落，携带与癌症相关的信息，在肿瘤精准治疗中有重要应用价值。脑脊液（cerebrospinal fluid, CSF）存在于大脑蛛网膜下腔、脊髓和中央管，与脑膜组织直接接触，是最能反映LM遗传特征的液体介质。近年来，解析CSF ctDNA多组学特征对深入探索中枢神经系统（central nervous system, CNS）转移瘤的辅助诊断、疗效判断、预后预测及耐药机制分析等具有重大意义。本文将对CSF ctDNA检测技术及其在NSCLC-LM患者中的临床应用进行简要综述。

## Abstract (en)

Leptomeningeal metastasis (LM) is a lethal complication of malignant tumors, with an incidence rate of 3%-5% among patients with non-small cell lung cancer (NSCLC). LM poses significant challenges in diagnosis, has poor prognosis, limited treatment options, and lacks standardized criteria for evaluating therapeutic efficacy, making it a difficult aspect of NSCLC management. Circulating tumor DNA (ctDNA), shed from tumor cells and carrying cancer-related information, holds significant value in precision oncology. Cerebrospinal fluid (CSF), present in the subarachnoid space of the brain, the spinal cord, and the central canal, and in direct contact with meningeal tissues, serves as the fluid medium that best reflects the genetic characteristics of LM. In recent years, CSF ctDNA has become a focal point due to its multi-omics features, playing a crucial role in the management of central nervous system (CNS) metastatic tumors. Its applications span the entire continuum of care, including aiding in diagnosis, assessing treatment response, predicting prognosis, and analyzing resistance mechanisms. This article provides a concise overview of CSF ctDNA detection techniques and their clinical applications in patients with NSCLC-LM.

人类的脑膜分为三层，从外到内分别是：硬脑膜、蛛网膜和软脑膜，蛛网膜和软脑膜共称为柔脑膜。柔脑膜转移（leptomeningeal metastasis, LM）是指肿瘤细胞扩散至软脑膜和蛛网膜，并通过蛛网膜下腔在脑脊液（cerebrospinal fluid, CSF）中播散。研究^[1,2]^发现有1%-5%的实体瘤患者会发生LM，以肺癌、乳腺癌、恶性黑色素瘤最为常见，特别是伴有表皮生长因子受体（epidermal growth factor receptor, EGFR）突变的NSCLC患者，发生率高达9%-16%。LM患者自然生存期短，未经治疗患者的中位生存期仅为4-6周^[3]^。肿瘤的病理学诊断和分子分型大多依赖组织活检，而LM病灶相对较小且脑膜活检创伤极大，几乎没有穿刺活检的可能。此外，癌症分子会随着时间推移而不断演变，复发时的基因组特征与首次诊断时存在差异^[4]^，单次组织活检提供的信息有限。液体活检相较于传统组织活检创伤小、可重复性强，有助于动态实时追踪癌症分子^[5]^。循环肿瘤DNA（circulating tumor DNA, ctDNA）是其中一个新兴热点，且广泛应用于肺癌领域^[6]^。ctDNA携带遗传信息和表观遗传信息^[7]^，包括基因突变、拷贝数变异（copy number variation, CNV）、微卫星不稳定以及甲基化修饰改变等，可揭示肿瘤基因组概况^[8,9]^。血液是最常用的液体活检样本，适用范围广。然而，与颅外病灶不同，中枢神经系统（central nervous system, CNS）转移瘤释放的DNA难以透过血脑屏障到达外周循环系统，血浆ctDNA反映颅内病灶分子特征的能力有限^[10,11]^。CSF与脑膜直接接触，且不易受到原发灶或其他颅外转移灶DNA干扰，是最能代表LM的液体介质。本文将对CSF ctDNA在NSCLC-LM诊断中的优势及局限、耐药机制分析、监测病情变化及预测预后等方面应用进行阐述。

## 1 CSF ctDNA来源、性质及相关检测技术

细胞游离DNA（cell-free DNA, cfDNA）是细胞坏死后释放到血液或其他体液（CSF、尿液、胸腔积液、胆汁等）中的DNA片段。cfDNA的发现最早可追溯到1948年，Mandel等^[12]^首次报道了健康人外周血中的cfDNA。研究^[13]^表明癌症患者体内cfDNA高于正常人，肿瘤来源的cfDNA被称为ctDNA。ctDNA易获取、半衰期短，与原发灶基因信息一致性高，是肿瘤负荷的可靠生物标志物^[14]^，正逐步应用于肿瘤诊疗的各个阶段。然而，外周血来源的ctDNA对CNS恶性肿瘤并不敏感^[10,11,15]^，Bettegowda等^[16]^的研究发现神经胶质瘤患者血浆ctDNA检出率<10%。原因有二^[17]^，一方面，血脑屏障限制了颅内肿瘤细胞及其所释放的DNA进入外周循环系统；另一方面，血液中存在大量正常细胞、肿瘤原发灶以及其他颅外转移灶脱落的DNA，使CNS来源的ctDNA被稀释。相比之下，CSF与颅内特殊环境密切相通，且细胞稀缺（0-5个细胞/µL），突变检测时正常DNA的背景干扰较小^[11]^。因此，尽管CSF中cfDNA总量较低^[18]^（每毫升CSF含1.75 ng cfDNA，每毫升血液含8.19 ng cfDNA），但更能代表颅内病灶的基因谱特征。

随着ctDNA富集、测序技术的发展，ctDNA检测大致分为两类，一类是用于评估已知突变的靶向检测，另一类是旨在同时识别多个已知或未知突变的非靶向检测。靶向检测大多基于聚合酶链式反应（polymerase chain reaction, PCR），包括扩增受阻突变体系（amplification refractory mutation system, ARMS）技术、BEAMing（beads, emulsion, amplification, and magnetics）技术、微滴式数字PCR（droplet digital PCR, ddPCR）技术等。这类检测用于外周血ctDNA技术成熟、价格便宜、用时短、易于解读，缺点是不能多个基因同时进行检测，也不能检测复杂突变，以CSF作为样本的相关研究较少。Yang等^[19]^使用ARMS-PCR检测了30例NSCLC脑转移患者，ARMS-PCR技术在CSF和原发灶之间检测出EGFR突变的敏感性为67%（95%CI: 0.36-0.97），特异性为82%（95%CI: 0.59-1.00），CSF中存在EGFR突变的患者通过EGFR-酪氨酸激酶抑制剂（tyrosine kinase inhibitors, TKIs）治疗取得了生存获益，表明ARMS-PCR是检测CSF中EGFR突变的敏感方法，可应用于NSCLC颅内转移患者的突变检测。有研究^[20]^通过ddPCR技术检测黑色素瘤LM患者的CSF，结果显示细胞学阴性的患者中，30%患者的CSF ctDNA呈阳性，提示ddPCR在分析CSF方面优于细胞学。近年来，由于NSCLC推荐检测靶点不断增加，只做一个或几个基因的检测无法满足临床需求。由下一代测序（next-generation sequence, NGS）技术所演化出的基因panel是现阶段临床中常用的方式，可针对靶向区域进行测序，覆盖多种基因，涵盖突变、CNV、基因融合等不同的变异形式，无论样本是外周血还是CSF。非靶向检测同样基于NGS技术，如全基因组测序（whole-genomic sequencing, WGS）和全外显子组测序（whole-exome sequencing, WES），其优势在于无需原发肿瘤基因组的信息，并能够发现未知的新型突变，但成本高昂，无法在临床中全面推广。

判断CSF ctDNA是否阳性大多依赖基因panel或ddPCR检测热点基因，如：EGFR、间变性淋巴瘤激酶（anaplastic lymphoma kinase, ALK）、间质上皮细胞转化因子（mesenchymal epithelial transition factor, MET）等。然而并非所有患者都具有驱动基因突变，野生型患者可通过WGS检测cfDNA中的体细胞拷贝数变异（somatic copy number alteration, SCNA）并使用ichorCNA工具^[21]^定量计算肿瘤分数（tumor fraction, TFx），即cfDNA中肿瘤来源DNA占比，类似于肿瘤组织中“肿瘤纯度”的概念，来判断CSF ctDNA是否存在。研究^[22]^证实TFx与等位基因变异丰度（mutant allele fraction, MAF）存在显著相关性。TFx>0.1是常用诊断界值，最佳cut-off值仍需要更大规模的前瞻性研究评估。

## 2 CSF ctDNA在NSCLC-LM诊断中的优势及局限

过去几年，随着肺癌靶向治疗和免疫治疗的不断发展和广泛应用，NSCLC患者的生存期大幅度延长，但不可否认的是LM的发病率也逐年上升^[23]^，早期、准确诊断LM对患者预后至关重要。NSCLC-LM的诊断是在肺癌确诊基础之上，基于临床表现、影像学检查、CSF细胞学检查进行综合评判。症状方面，LM的表现复杂多样，缺乏特异性，如：头痛、恶心、呕吐、视觉障碍、听力受损等，临床上容易误诊和漏诊。影像学方面，首选的检查是磁共振成像（magnetic resonance imaging, MRI），可以显示脑膜受累的直接征象。然而，MRI对LM的敏感性为70%-85%，特异性为75%-90%^[23]，约30% LM患者MRI无异常表现[2]^^，临床中主要起提示作用^。CSF细胞学作为诊断LM的金标准，首次取样时的阳性率为40%-60%，重复取样时才能将阳性率提高至80%左右^[24]^。考虑到LM的快速进展和不良预后，临床迫切需要找到更加敏感的诊断方法。

CSF ctDNA是一种极具诊断潜力的生物标志物，与细胞学检查、影像学相比敏感性更高^[25]^。Zhang等^[17]^招募了21例NCSLC-LM患者，收集每例患者的CSF，分别进行基因检测和病理细胞学检查，结果显示CSF ctDNA阳性率为100%（21/21），细胞学阳性率仅为81%（17/21），CSF ctDNA检测的灵敏性在LM诊断方面优于细胞学（P<0.001）。一项小型队列研究^[26]^也得到了相似的结论，研究者使用低深度全基因组测序（shallow whole-genomic sequencing, sWGS）检测LM患者的CSF，结果发现CSF ctDNA敏感性高于CSF细胞学检查（93% vs 72%, P=0.02）。

然而，以上研究均是在研究对象已确诊LM的前提下，比较不同方式之间的敏感性，无法作为CSF ctDNA可用于诊断LM的直接证据。其局限性在于部分脑实质转移灶同样能释放ctDNA进入CSF，干扰LM的诊断。数据^[27]^表明约有30.7%的LM患者合并脑实质转移，对于脑实质转移与LM的鉴别，CSF ctDNA的表现并不理想。Michael等^[26]^对3例仅有脑实质转移患者的CSF进行sWGS，结果均为阳性。Li等^[28]^的研究发现，颅内病灶越大、离脑室越近，检测到CSF ctDNA的概率越高。总之，CSF ctDNA对NSCLC-LM敏感性高、特异性低的特点使得它目前无法替代CSF细胞学检查在诊断上的地位，而对于无脑实质转移且疑似LM的NSCLC患者，是否可以利用CSF ctDNA作为诊断标准之一仍需要临床进一步验证。

为了更好地区分脑实质转移和LM，有研究^[29]^尝试对CSF ctDNA与CSF癌胚抗原（carcinoembryonic antigen, CEA）进行组合分析，阳性定义为CSF CEA或CSF ctDNA阳性，纳入了39例LM患者、15例非LM患者（8例有脑实质转移，7例无脑实质转移），其中联合方法的敏感性和准确性分别为0.97（95%CI: 0.87-1.00）和0.94（95%CI: 0.85-0.99），与单一CSF ctDNA检测相比有显著差异（P<0.01）。以上结果虽然不能彻底解决CSF ctDNA的局限性，但为区分LM和脑实质转移提供了方向。未来，标准化的临床症状评价和神经影像学检查与CSF肿瘤细胞衍生物质检测相结合将改善NSCLC-LM的诊断现状。

## 3 CSF ctDNA可用于NSCLC患者TKIs的耐药机制分析

虽然驱动基因阳性的NSCLC患者在接受TKIs治疗后疗效良好，但是大多数患者会在与药物的“蜜月期”后产生耐药性。脑膜是TKIs耐药后的常见进展部位之一^[23]^。不仅如此，LM对NSCLC患者生存期的影响高于颅外病灶^[30]^，所以明确颅内耐药机制并更换相应的靶向药物十分重要。

由于CNS的特殊环境以及颅内外病灶对药物的敏感性不同^[31]^，LM的耐药机制与颅外病灶之间存在差异。在Wu等^[32]^的研究中，对1例接受EGFR-TKIs治疗后出现LM的患者进行基因检测，结果显示CSF和外周血均检测到EGFR C797S突变，但它们具有不同的核苷酸变体，在外周血中表现为EGFR Cis T790M C797S（2390G>C, 0.5%），而在CSF中表现为EGFR C797S（2389T>A, 8.8%），表明两种耐药突变分别由颅内外病灶独立进化而来。此外，Li等^[33]^报道了3例NSCLC-LM患者在症状恶化后完善CSF基因检测，发现EGFR 20号外显子插入、c-ros肉瘤致癌因子-受体酪氨酸激酶（ROS proto-oncogene 1, receptor tyrosine kinase, ROS-1）突变、MET扩增等新发突变，予换药治疗后取得显著疗效。因此，对于TKIs治疗后出现颅内进展的NSCLC患者，有必要通过CSF明确颅内转移瘤基因突变情况，并指导下一步治疗。

EGFR T790M是CNS获得性耐药的最常见原因之一^[34]^，但有文献^[35⇓⇓-38]^提示CSF中T790M突变的检出率远低于外周血。研究者推测是血脑屏障限制TKIs渗透到CNS，药物无法在颅内达到有效浓度，导致CSF中继发性T790M耐药突变相对较少^[17,39]^，这为常规剂量TKIs治疗失败后加倍剂量再挑战提供了理论基础。在Huang的研究中^[40]^，有患者接受第一代EGFR-TKIs治疗后出现颅内进展，CSF基因检测提示EGFR T790M阴性、EGFR 21L858R阳性，并予第一代药物加量治疗，患者最终疾病稳定长达12个月。Zheng等^[41]^对81例奥希替尼治疗失败且进展部位为LM的患者进行CSF基因检测，其中14例未检测到已知耐药靶点的患者接受了奥希替尼加量治疗，该队列中位总生存期为12.5个月，获得了生存受益。

另外，CSF ctDNA中大量独特的CNVs可能是NSCLC-LM潜在转移和获得性耐药的分子机制^[17,32,39,42,43]^。常见CNVs有EGFR扩增、TP53杂合性丢失（loss of heterozygosity, LOH）、MET扩增、MYC扩增、RB1缺失、HER2扩增等。在既往文献中，MET扩增可导致患者对吉非替尼耐药^[44]^，MET扩增与肿瘤细胞适应颅内微环境相关，促进肿瘤细胞在颅内生存^[45]^，TP53 LOH和RB1缺失是小细胞肺癌转化的分子特征，可导致NSCLC患者产生耐药^[46]^，EGFR扩增是EGFR-TKIs耐药的潜在途径^[10]^。总体而言，CSF ctDNA检测有利于了解NSCLC-LM患者分子分型，并进行疗效及耐药监测，最终实现肿瘤个体化精准治疗。

## 4 CSF ctDNA可用于NSCLC-LM患者疗效评价及预测预后

目前实体瘤的疗效评价多依赖影像学检查测量的肿瘤直径的动态变化，比如实体瘤疗效评价标准（Response Evaluation Criteria in Solid Tumors, RECIST）及其衍生的实体瘤免疫治疗疗效评价标准（immune-modified RECIST, iRECIST）^[47]^。然而，这类评效方法无法早期捕捉到肿瘤进展，难以判断假进展和疾病稳定的患者是否有响应，且不确定性因素较多，如：靶病灶选择、阅片者资质和经验、设备差异等。

ctDNA半衰期短，<2 h^[48]^，能克服肿瘤的时间及空间异质性^[49]^，能够动态反映肿瘤负荷变化^[9,21,50]^，有望作为疗效评价的候选生物标志物。有研究^[14]^回顾了液体活检在评估实体瘤疗效中的应用，并提出了液体活检反应实体瘤评价标准（Liquid Biopsy Response Evaluation Criteria in Solid Tumors, LB-RECIST）标准化的计划。

与实体瘤不同，LM多为点状或线状强化，无法测量，因此对LM患者的疗效评估是一项极具挑战性的课题。现阶段没有统一的LM疗效评价标准，不同的研究基于不同的评价标准，很难相互比较治疗方式的有效性。临床上最具权威的是RANO-LM标准^[51]^，但研究^[52]^表明，RANO-LM标准在临床实践中面临困难，表现为临床一线医生很难掌握该标准且不同医生对同一患者评价的一致性低。研究^[17]^发现，CSF ctDNA中驱动基因突变丰度与临床症状及影像学检查并行，有时甚至提前于影像学变化。鉴于目前仍有许多可行性和临床适用性相关问题需要解决，因此开展有关CSF ctDNA评效标准的研究势在必行。

染色体不稳定性会导致DNA丢失、增加和重排，使肿瘤不断产生新的基因变异，推动肿瘤进展^[53]^。CSF ctDNA中CNVs的高发生率表明LM中普遍存在染色体不稳定性^[54]^。染色体不稳定性为判断NSCLC-LM患者预后提供了可行手段。Wu等^[32]^的研究通过低深度-WGS（low-coverage WGS, LC-WGS）技术，用区段染色体断点数量（large-scale transitions, LSTs）量化染色体不稳定性。对24例NSCLC-LM患者随访发现，高水平LSTs患者（≥15.5）生存期明显短于低水平LSTs患者（<15.5），提示LSTs是潜在的预后标志物。Wang等^[55]^建立了染色体不稳定性评分来描述NSCLC-LM患者CSF中的染色体不稳定性状态，评分高的患者（>0.07）与不良预后、高颅压、低卡氏体力状态（Karnofsky performance status, KPS）评分相关，且无LM生存期和总生存期更短（1.7 vs 2.9年，P=0.021；2.5 vs 3.9年，P=0.038）。可见CSF ctDNA中染色体不稳定性在预测NSCLC-LM患者预后方面表现出独特优势。

## 5 CSF ctDNA具有独特的基因谱

CSF ctDNA为NSCLC-LM患者提供了更全面的基因信息，包括驱动基因突变、伴随基因突变、独特的CNVs等。

### 5.1 驱动基因

CSF ctDNA在识别NSCLC LM驱动基因方面优于外周血ctDNA，特别是EGFR和ALK^[39,42,56]^。Yang等^[10]^招募了34例NSCLC-LM患者，比较CSF和配对血的驱动基因组突变谱，结果显示CSF中驱动基因的检出率和等位基因突变频率均显著高于配对血。Zhang等^[17]^的研究对21例EGFR阳性NSCLC-LM患者的CSF和配对血进行基因检测，其中CSF的EGFR检出率为100%，血液仅为52%（P<0.001），CSF中EGFR的平均突变丰度为22.79%，外周血仅为0.57%（P<0.001）。因此，对于颅内转移患者，外周血或组织样本基因检测阴性时，应当进一步完善CSF ctDNA检测，提高临床可操作突变检出率。

### 5.2 伴随基因

NSCLC-LM患者CSF中有大量伴随基因，如TP53、RB1、PIK3CG、CDK4、CDK6、CDKN2A、FGFR3、FGFR4、PIK3R1、AKT3、STK11、CTNNB1、PMS2等。其中TP53、RB1是CSF中最常见的伴随基因^[57]^，CDKN2A/B、CDK4/6、PIK3CA/G仅见于CSF^[10]^，且均与不良预后相关。FGFR3、FGFR4、PIK3R1、AKT3、STK11与PI3K/Akt/mTOR通路相关，该通路激活可能与LM的发生有关^[17]^。CTNNB1属于Wnt信号通路，Wnt通路在肺癌转移至脑组织的过程中发挥关键作用，PMS2与DNA错配修复蛋白缺失（deficiency of MMR, dMMR）有关^[54]^。此外，CSF中更易检出EGFR/RB1、TP53/EGFR、TP53/RB1、TP53/ERBB2、TP53/KMT2C等共突变事件，具有以上共突变的患者预后更差^[55]^。

### 5.3 CNVs

对于EGFR、ALK以外的驱动基因，CSF中突变类型大多为CNVs，而非靶向治疗中常见的融合或重排，包括MET扩增、ERBB2扩增、BRAF扩增、NTRK1扩增^[56,57]^。TP53 LOH在CSF中的检出率显著高于外周血。与此同时，具有TP53 LOH突变的患者基因谱更加复杂^[43]^，表现为伴随更多EGFR、MET、RB1基因突变及CNVs^[56]^，这种情况是CSF独有的。考虑到CNVs在肿瘤转移过程中起重要作用，因此未来有必要探究这些基因出现CNVs的意义。

## 6 结语与展望

NSCLC患者LM发生率逐年提高，与靶向治疗的进展、MRI的广泛使用、患者生存期延长以及临床医生的逐步重视有关。寻找用于NSCLC-LM的微创检测技术及脑转移组织的液体代替标本一直是一项艰巨的任务，CSF ctDNA检测因其在诊断、指导治疗、检测耐药、监测疗效、预测预后等领域的价值与潜力而受到广泛关注。

尽管如此，CSF ctDNA仍面临着诸多限制。首先，ctDNA检测价格昂贵且十分耗时，相比之下细胞学检查可以在更短时间内完成。第二，在临床实践中，许多患者可提取到的CSF cfDNA量不能满足质控要求，导致检测结果可信度低。未来需要更灵敏的检测方法和更先进的生物信息学技术，从而拓展液态活检的适用人群。第三，CSF ctDNA检测对NSCLC-LM免疫治疗指导作用有限。现阶段肺癌治疗已进入免疫时代，CSF中也存在相关免疫反应^[58]^，期待CSF ctDNA在驱动基因阴性NSCLC-LM患者免疫治疗中的应用得到探索和拓展。

综上所述，单一的基因组学分析很难揭示NSCLC-LM发生发展的全貌。将CSF ctDNA与转录组学、代谢组学、蛋白质组学相结合进行多组学整合分析，有助于加深对NSCLC-LM的理解，提供更加准确的信息。相信，随着相关技术的不断完善，CSF ctDNA能在NSCLC-LM诊疗方面实现进一步突破，指导临床治疗方案制定，真正实现个体化精准医疗。
